# Topologically associating domains of chromatin on single-cell Hi-C data: a survey of bioinformatic tools and applications in the light of artificial intelligence

**DOI:** 10.3389/fgene.2025.1602234

**Published:** 2025-07-01

**Authors:** Hongqiang Lyu, Yao Li, Xinran Chen, Yuan Liu, Erhu Liu, Xiaoliang Cheng

**Affiliations:** ^1^ School of Automation Science and Engineering, Faculty of Electronic and Information Engineering, Xi’an Jiaotong University, Shaanxi, China; ^2^ School of Information and Control Engineering, Xi’an University of Architecture and Technology, Shaanxi, China; ^3^ Department of Pharmacy, The First Affiliated Hospital of Xi’an Jiaotong University, Shaanxi, China

**Keywords:** topologically associating domains, single-cell Hi-C, bioinformatics tools and applications, artificial intelligence, challenges and emerging trends

## Abstract

Topologically associating domains (TADs) uncovered on bulk Hi-C data are regarded as fundamental building blocks of a three-dimensional genome, and they are believed to effectively participate in the regulatory programs of gene expression. The computational analysis of TADs on single-cell Hi-C (scHi-C) data in the era of single-cell transcriptomics has received continuous attention since it may provide information beyond that on bulk Hi-C data. Unfortunately, the contact matrix for a single cell is ultra-sparse due to the low sequencing depth. Coupled with noises, artifacts, and dropout events from experiments, as well as cell heterogeneity caused by the cell cycle and transcription status, the computational analysis of TAD structures at the single-cell level has encountered some challenges not encountered at the bulk level. Herein, conduct a survey of bioinformatic tools and applications for TAD structures at the single-cell level in the light of artificial intelligence, including imputation of scHi-C data, identification of TAD boundaries and hierarchy, and differential analysis of TAD structures. The categories, characteristics, and evolutions of the latest available methods are summarized, especially the artificial intelligence strategies involved in these issues. This is followed by a discussion on why deep neural networks are attractive when discovering complex patterns from scHi-C data with an enormous number of cells and how it promotes the computational analysis of TADs at the single-cell level. Furthermore, the challenges that may be encountered in the analysis are outlined, and an outlook on the emerging trends in the near future is presented cautiously.

## 1 Introduction

### 1.1 What are topologically associating domains?

Topologically associating domains (TADs) are chromatin regions that show a high degree of self-interactions between loci within the domains and a low degree of interactions with loci outside the domains, even if they are a similar distance away (Dixon et al., 2012; Bonev and Cavalli, 2016). The TAD structures were discovered in 2012 (Dixon et al., 2012) with the help of the high-throughput chromosome conformation capture (Hi-C) technology (Lieberman-Aiden et al., 2009) at the bulk level. Hi-C is a powerful experimental technology that combines high-throughput sequencing with chromosome conformation capture (3C) (Dekker et al., 2002), enabling the profiling of chromatin spatial interactions on a genomic scale. It produces up to billions of paired-end reads at the bulk level, followed by mapping, fragment assignment, filtering, and binning procedures, and a contact matrix, which is also known as an interaction matrix, is generated. In a contact matrix, each row or column is called a bin representing a fixed-size segment of DNA along chromosomal coordinates. The fixed length is referred to as resolution, with smaller values indicating higher resolutions. Each element in the matrix indicates the frequency at which two bins physically associate in the 3D space of chromatin. Its value is known as the interaction frequency, which exhibits an exponential decay with an increase in the distance between the two bins. Owing to the Hi-C contact matrix, the spatial organizations of chromatin on multiple scales have been investigated, including A/B compartments (Lieberman-Aiden et al., 2009), TADs (Dixon et al., 2012), and chromatin loops (Rao et al., 2014). Among them, TADs have gained much attention since they are the fundamental structural units of chromatin organizations and important functional units of gene regulations. Getting off the ground, TADs were regarded as the triangular blocks of elevated interaction frequencies along the diagonal of the contact matrix (Dixon et al., 2012). These blocks are considered self-contained regulatory circuits, underscoring their critical role in maintaining insulated regulatory landscapes (Dixon et al., 2012). Further studies showed that TADs exhibit a hierarchical architecture; that is, sub-TADs are nested within meta-TADs. The hierarchy balances structural stability with functional plasticity, which means that while the meta-TADs preserve overall domain integrity across cell types, the nested sub-TADs undergo dynamic reorganization during cellular differentiation to facilitate cell state-specific transcriptional programs. This structural adaptability enables precise spatiotemporal control of gene regulation while maintaining genomic stability (Berlivet et al., 2013; Phillips-Cremins et al., 2013).

TAD structures have received continuous attention in the era of single-cell transcriptomics. Unlike the bulk Hi-C technology, which captures population-averaged spatial interactions of chromatin, single-cell Hi-C (scHi-C) technology was put forward in 2013, which allows for the profiling of spatial interactions of individual cells, leading to the examination of cell-to-cell variability in chromatin organizations (Nagano et al., 2013). The initial protocol of scHi-C was refined by the same team in 2017 to improve its throughput and sensitivity (Nagano et al., 2017). Almost at the same time, a single-nucleus Hi-C technology that can provide tenfold more contacts per cell than the initial protocol was used (Flyamer et al., 2017). Throughout the development of scHi-C and its follow-up derivative technologies, TAD structures have always been in focus, such as an investigation on whether TAD structures exist at the single-cell level, similar to that at the bulk level. Until 2018, stochastic optical reconstruction microscopy was employed to generate the 3D image of chromatin in numerous pseudocolors, reporting the positions and structures of a 30-kb segment with nanoscale precision. The imaging data demonstrate that TAD-like domains are physical structures with spatially segregated globular conformations at the single-cell level, and the positions of their boundaries show cell-to-cell variations (Bintu et al., 2018). In 2022, a new technology called scSPRITE achieved higher-resolution maps of individual cells than that can be produced by proximity use of ligation since it can measure multiway DNA contacts by introducing split-and-pool barcoding into the scHi-C protocol. With the support of scSPRITE, TAD structures were investigated on thousands of mouse embryonic stem cells, which helped reveal chromatin organizations that govern regulatory programs, offering deep insights into lineage commitment across cell populations (Arrastia et al., 2022). Overall, these experiments at the single-cell level show that TADs are not merely statistical artifacts reflecting population-level interaction tendencies but instead represent intrinsic characters of chromatin organizations of individual cells. Under the guidance of these experiments, currently, some computational methods have been used for the analysis of TAD structures on scHi-C data. Among them, a few of the latest methods have even started to dissect the hierarchy of TADs, although it remains an open question on whether TAD structures are also hierarchized at the single-cell level.

### 1.2 Computational analysis of TADs on scHi-C data

**FIGURE 1:**
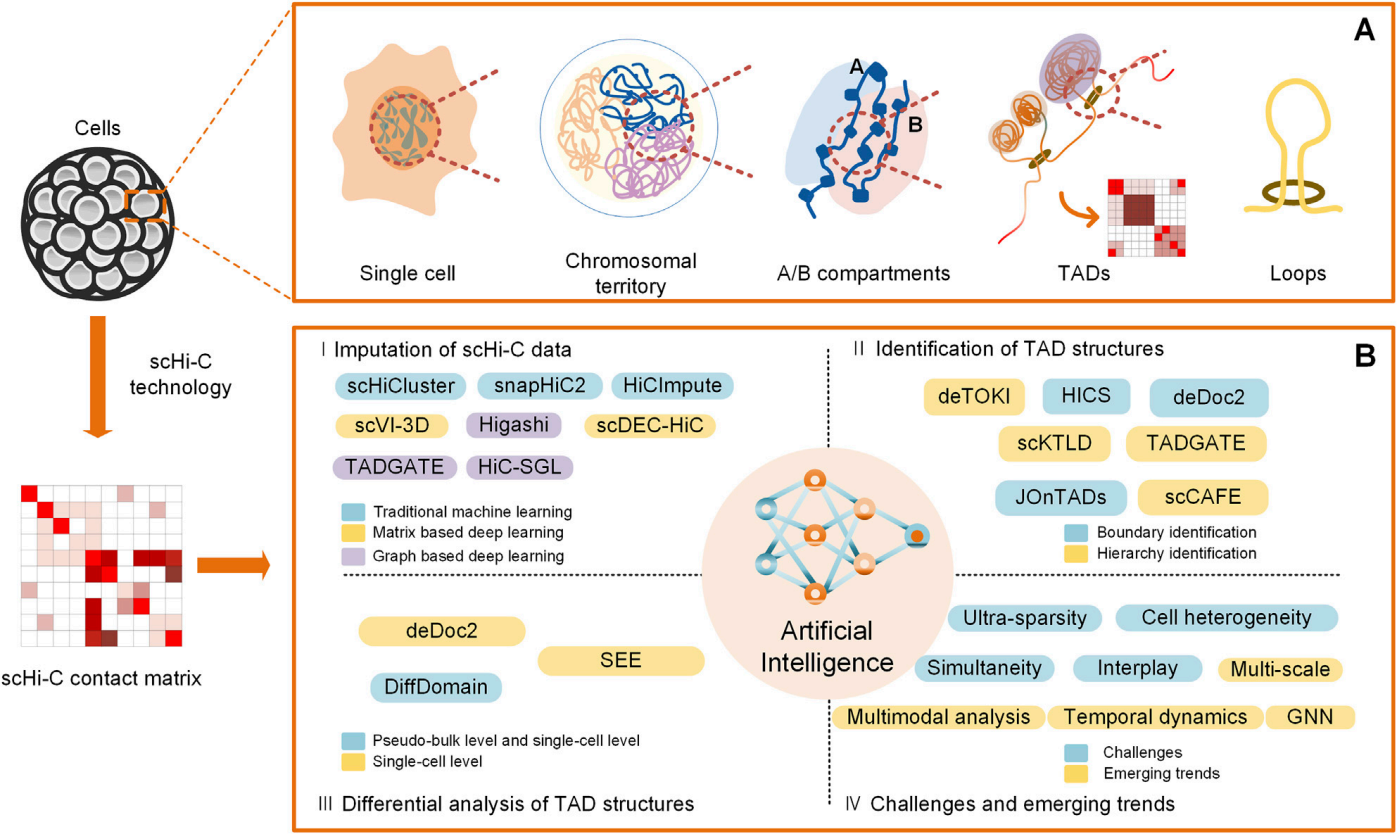
Overview of computational analysis for TADs on scHi-C data. **(A)** Spatial organizations of chromatin on multiple scales, including A/B compartments, TADs, and loops. **(B)** Computational methods for analysis of TADs at the single-cell level in the light of artificial intelligence, including imputation of scHi-C data, identification of TAD structures, differential analysis of TAD structures, and challenges and emerging trends.

Keeping in step with the experimental explorations of chromatin organizations of individual cells, similar to that at the bulk level, some state-of-the-art bioinformatic tools and applications for the analysis of TAD structures at the single-cell level come into being. They span over a variety of regards; herein, we mainly focus on their advancements in terms of the imputation of scHi-C data, identification of TAD structures, differential analysis of TAD structures, and challenges and emerging trends in the light of artificial intelligence (Figure 1).

## 2 Imputation of scHi-C data

It has been observed that some sequencing read signals of individual cells may not be captured in scHi-C experiments due to its low sequencing depth, cross-linking efficiency differences, and biological variations (Zheng et al., 2022). That causes some interaction frequencies to be observed at a low or moderate count level in one cell but not be detected in another cell under the same population, which is known as the dropout event. The curse of these dropouts in scHi-C data inevitably hinders the downstream analysis of TAD structures. Thus, an imputation preprocessing is devoted to addressing this problem, which aims to separate the dropout zeros from biological zeros, and tries to recover the missing interaction frequencies caused by dropout events so that the overall quality of the scHi-C data can be enhanced, thus allowing researchers to make full use of the data and perform more accurate downstream analyses, such as TAD structures.

Currently, several imputation methods for scHi-C data have been developed. At the very beginning, considering that the imputation methods for scRNA-seq data have made breakthroughs in recent years, how well these methods can be applied to scHi-C data was investigated, including MAGIC (van Dijk et al., 2018), scImpute (Li and Li, 2018), SCRABBLE (Peng et al., 2019), and scRMD (Han et al., 2020). The results show that these imputation methods indeed have an ability to handle scHi-C data, but they are mainly evaluated via their impact on cell clustering, leaving other biologically meaningful concerns uninvolved (Han et al., 2020). Roughly around the same time, the methods dedicated to scHi-C data imputation have emerged (Table 1). According to the representation format of scHi-C data, these imputation methods can be divided into two distinct categories, namely, matrix imputation and graph imputation. The former directly runs on the scHi-C contact matrix, including HiCImpute (Xie et al., 2022), scVI-3D (Zheng et al., 2022), and scDEC-Hi-C (Liu et al., 2022). Meanwhile, the latter treats the contact matrix as the adjacency matrix of a graph, including scHiCluster (Zhou et al., 2019), SnapHiC2 (Li et al., 2022), Higashi (Zhang et al., 2022), TADGATE (Dang et al., 2024), and HiC-SGL (Zheng et al., 2024). In accordance with the model structure and learning paradigms, these methods can be grouped into traditional machine learning imputation and deep learning imputation. The former requires handcrafted model design and iterative optimization, including scHiCluster, SnapHiC2, and HiCImpute. The latter conducts a training on an end-to-end deep neural network with large-scale data, including scVI-3D, Higashi, scDEC-Hi-C, TADGATE, and HiC-SGL. Depending on the processing scope of scHi-C data, these methods can be partitioned into the chromosome scale and genome scale. The former focuses on one individual chromosome at a time, including scHiCluster, SnapHiC2, HiCImpute, scVI-3D, scDEC-Hi-C, and TADGATE. Meanwhile, the latter utilizes the information throughout the entire genome, including Higashi and HiC-SGL. In addition, according to whether external data are introduced, these methods can also be separated into monomodal imputation and multimodal imputation. The former relies solely on scHi-C data, including scHiCluster, SnapHiC2, scVI-3D, scDEC-Hi-C, TADGATE, and HiC-SGL. Meanwhile, the latter makes use of other types of data apart from scHi-C data, including HiCImpute for bulk Hi-C data and Higashi for epigenomic signals.

**TABLE 1:** Methods for imputation of scHi-C data.

Method	Group	Description	Language	Year
scHiCluster	Graph/CS	Random walk with restart	Python	2019
SnapHiC2	Graph/CS	Random walk with restart, sliding window	Python	2022
HiCImpute	Matrix/CS	Bayesian hierarchical model	Python	2022
scVI-3D	Matrix/DL/CS	Variational autoencoder (VAE)	Python	2022
Higashi	Graph/DL/GS	Hypergraph neural network	Python	2022
scDEC-Hi-C	Matrix/DL/CS	Generative adversarial network	Python	2023
TADGATE	Graph/DL/CS	Graph attention autoencoder	Python	2024
HiC-SGL	Graph/DL/GS	Subgraph extraction, graph representation learning	Python	2024

“CS” and “GS” denote the chromosome scale and genome scale, respectively. “DL” indicates deep learning.

In view of the advances and evolution along time, the first method devoted to the imputation of scHi-C data, called scHiCluster, came into being in 2019, where a strategy of random walk with restart is used on a graph since the scHi-C contact matrix can be naturally regarded as the adjacency matrix of an edge-weighted undirected graph due to its symmetry and ultra-sparsity at the single-cell level, with nodes corresponding to bins and interaction frequencies being the weights of edges. In 2022, several imputation methods for scHi-C data emerged: SnapHiC2 adopts a sliding window approximation to accelerate the graph imputation on the basis of scHiCluster. HiCImpute establishes a Bayesian hierarchical model to distinguish structural zeros from dropout zeros by leveraging bulk Hi-C data. scVI-3D pioneers a matrix imputation by introducing a deep generative model of the variational autoencoder. Higashi pushes the imputation of scHi-C data from a chromosome scale forward to a genomic scale using a hypergraph representation learning framework that incorporates scHi-C data and epigenomic signals. In 2023, scDEC-Hi-C emerged as a matrix imputation with the help of the generative adversarial network. In addition, in the year 2024, the graph neural network (GNN) mainly dominated this field, such as TADGATE with the graph attention autoencoder and HiC-SGL with subgraph extraction and graph representing learning. In the GNN, the imputation of scHi-C data can be regarded as a link prediction problem, where the lost edges between nodes can be predicted by leveraging other existing edges in the graph, and the propagation of information between neighboring nodes during the training of GNN can contribute to the prediction of lost edges. In simple terms, assuming that there is a strong link between nodes A and B via an edge and that nodes B and C are in the same case, it is very likely that nodes A and C are linked, even though the edge between them is unseen. That makes the graph neural network more preferable for imputation compared with matrix-based approaches. Generally, the imputation methods for scHi-C data are advancing and evolving from matrix imputation to graph imputation, from traditional machine learning to matrix-based neural networks and even graph neural networks, from the chromosome scale to genomic scale, and from the monomodal strategy to multimodal strategy.

## 3 Identification of TAD structures

The identification of TAD structures has always been an interesting issue from the time when it was found on Hi-C contact matrix at the bulk level since they are believed to be the fundamental structural units of chromatin organizations and their disruptions can be linked to various genetic disorders and cancers, providing essential insights into genomic function and disease mechanisms. With the rapid development of the scHi-C technology in recent years, whether TADs found at the bulk level can also be detected on the scHi-C contact matrix has become a concern. It has been demonstrated that the bioinformatic methods designed for the identification of TADs at the bulk level are not applicable on scHi-C data due to the ultra-sparsity of the contact matrix, noises, and artifacts from experiments and heterogeneity of individual cells. More specifically, the proportion of zeros in the scHi-C contact matrix is extremely large, and the interaction frequencies are usually quite small as a result of the low sequencing depth. Moreover, these weak interaction signals are coupled with various noises and artifacts introduced during experiments and are diversified by different functional states of individual cells, such as cell cycle and transcription status. Thus, the boundaries between TADs are not apparent, and the interaction patterns within them are blurred.

At present, several bioinformatic methods have been proposed for the identification of TAD structures on the scHi-C contact matrix, imputed or not (Table 2). According to whether the hierarchical architecture of TADs is considered, these methods can be divided into boundary identification and hierarchy identification. The former regards TADs as insulated regions without mutual containing, including deTOKI (Li et al., 2021), our scKTLD (Liu et al., 2024), TADGATE (Dang et al., 2024), MUTI (Zhou et al., 2024), and scCAFE (Wang et al., 2024). Meanwhile, the latter takes into account the sub-TADs nested within meta-TADs, including deDoc2 (Li et al., 2023), HiCS (Ye et al., 2023), and JOnTADs (Zeng et al., 2024). Depending on the representation format of scHi-C data, these methods can also be grouped into identification by matrix and identification by graph. The former regards TADs as blocks that are symmetric along the diagonal of the contact matrix and has higher interaction frequencies, including deTOKI, HiCS, deDoc2, MUTI, and JOnTADs. Meanwhile, the latter treats TADs as communities of nodes within a graph that have a stronger density of connections, including our scKTLD, TADGATE, and scCAFE. In addition, as far as we know, MUTI is the only multimodal approach that conducts an analysis at the TAD level by integrating non-simultaneous scHi-C and scRNA-seq data. Only scCAFE has the ability to identify the spatial organizations of chromatin on multiple scales; i.e., apart from TADs, it can also detect A/B compartments and loops simultaneously at the single-cell level, while the other methods remain on a single scale.

**TABLE 2:** Methods for identification of TAD structures on scHi-C data.

Method	Group	Description	Language	Year
deTOKI	Boundary/matrix	Non-negative matrix factorization	Python	2021
HiCS	Hierarchy/matrix	Find peaks of insulation strength at different levels	Python	2023
deDoc2	Hierarchy/matrix /DA	Dynamic programming, minimum structural entropy Mann–Whitney U test	Java	2023
scKTLD	Boundary/graph	Graph embedding, change point detection	Python	2024
TADGATE	Boundary/graph	Graph attention autoencoder	Python	2024
MUTI	Boundary/matrix/multimodal	Insulation score	Python	2024
JOnTADs	Hierarchy/matrix /multi-sample	Line-shaped scanning and dynamic programming	Python	2024
scCAFE	Boundary/graph /multi-scale	Graph VAE and hierarchical clustering with connectivity constraints	Python	2024
DiffDomain	Boundary/matrix /DA	High-dimensional random matrix theory	R/Python	2024
SEE	Matrix/DA	Interaction density variation mapping	Python	2025

“DA” indicates differential analysis.

From the view of the timeline, the identification of TAD structures at the single-cell level is pioneered by deTOKI that was launched in 2021. This method splits the scHi-C contact matrix into sub-matrices and employs non-negative matrix factorization to seek the regions that insulate the chromatin into blocks with minimal chance of clustering. Since 2023, HiCS and deDoc2 pushed the identification of TAD boundaries forward to that of the hierarchical architecture at the single-cell level. The former converts the problem of the identification of hierarchical TADs into finding peaks of insulation strength at different levels, and the latter employs a dynamic programming strategy to find chromatin partitions with global minimal structure entropy for both the whole and local matrix. In 2024, several methods rapidly emerged. Our scKTLD and TADGATE began to identify TAD structures by graph rather than by matrix. The former introduced graph embedding and change point detection to discover TAD boundaries. Meanwhile, the later turns to the graph neural network, a graph autoencoder specifically, to detect boundaries. MUTI identifies TAD boundaries using an insulation score and conducts a further analysis at the TAD level by introducing a multimodal strategy relying on scHi-C and scRNA-seq data. In addition, there were two other methods that were launched in the same year, including JOnTADs and scCAFE. JOnTADs still calls TAD boundaries by the matrix instead of by graph using line-shaped scanning and dynamic programming. Nevertheless, it is applicable on the Hi-C contact matrix at both bulk and single-cell levels, and it has the ability to handle multiple samples. In addition, as the latest method in this year, scCAFE builds a unified framework for the identification of chromatin spatial organizations on multiple scales, including A/B compartments, TADs, and loops. Among them, TAD boundaries are detected by graph VAE and hierarchical clustering with connectivity constraints. It can be seen that these methods generally exhibit a progression from the TAD boundary to TAD hierarchy, from the matrix to graph, from traditional machine learning to the graph neural network, and from only TADs to multi-scale architectures.

## 4 Differential analysis of TAD structures

Differential analysis has always been a long-standing topic in bioinformatic analysis of diverse omics data at the single-cell level since it allows the investigation of cell heterogeneity, cell differentiation, and the occurrence and progression of diseases. The TAD structures of individual cells are dynamically changing and are closely related to the spatial regulation of gene expression. For example, at the single-cell level, a significant decrease in TAD structure strength has been observed during the transition from transcriptionally active immature oocytes to transcriptionally inactive mature oocytes in mice (Flyamer et al., 2017). In addition, the disruption of TAD boundaries has also been discovered in malignant glioblastoma cells, which can rewire enhancer–promoter interactions and contribute to glioblastoma progression (Chang et al., 2024). This makes the differential analysis of TAD structures helpful for discovering structural changes of chromatin organizations across different conditions. The structure affects the function, and the differential analysis of TAD structures is beneficial for gaining new biological insights and understanding how the 3D genome organizations are disrupted in diseases.

The computational differential analysis of TAD structures on scHi-C data is still in the early stage of development. Due to the ultra-sparsity of the scHi-C contact matrix and heterogeneity of individual cells, differential analysis of TADs is gradually moving ahead in the midst of various difficulties and challenges, and only a few methods have emerged recently, including deDoc2 (Li et al., 2023), DiffDomain (Hua et al., 2024), and SEE (Li et al., 2025). Among them, deDoc2 and SEE are designed for TADs at the single-cell level. The former gives out the TAD structure changes between cell-cycle stages of mice embryonic stem cells using the Mann–Whitney U test beyond the identification of hierarchical TADs. In addition, the latter reveals the rearrangements of TADs during differentiation of oligodendrocyte cells by interaction density variation mapping, while conducting an investigation of chromatin dynamics on the combination of scRNA-seq data and scHi-C data. DiffDomain was initially designed for differential analysis of TADs at the bulk level and can be extended to Hi-C data at the single-cell level using a pseudo-bulk strategy. Moreover, it is advantageous that DiffDomain has the ability to separate the TAD structural variations into six distinct groups, namely, strength-change, loss, split, merge, zoom, and complex patterns, and it can also quantify the cell-to-cell variability of TAD structures directly on the scHi-C contact matrix imputed by scHiCluster. Generally, it seems that the deep learning network dedicated to differential analysis of TAD structures at the single-cell level has not been formed yet. There is still a lot of room for development in the field.

## 5 Artificial intelligence in TAD analysis

The introduction of artificial intelligence benefits the bioinformatic analysis of TADs at the single-cell level, considering a great number of cells from scHi-C experiments and the complex patterns underlying the large-scale scHi-C data. Getting off the ground, some traditional methods that are successful on sparse matrices and other single-cell omics data were introduced into the analysis of TADs at the single-cell level, such as the random walk with restart strategy in scHiCluster, which is the first method for scHi-C data imputation, and the non-negative matrix factorization algorithm in deTOKI, which is the first method for TAD identification at the single-cell level. These traditional methods have some advantages, such as a clear decision-making process, less dependence on a large sample size, and incorporation of prior knowledge. However, while deep learning methods were introduced into the analysis of TADs on scHi-C data, their outstanding performance is truly impressive. It was observed that the end-to-end deep learning network typically surpasses traditional methods, owing to its advantage of learning knowledge from large-scale scHi-C data coupled with noises and artifacts.

The artificial intelligence methods for TAD analysis at the single-cell level are evolving. scHi-C data are usually presented in the form of a contact matrix, and a matrix-based neural network is relatively mature, which makes the matrix-based deep learning methods involved first, such as the variational autoencoder in scVI-3D. With time, the limitations of this kind of deep learning methods have been gradually exposed. They accept fixed-size input tensors, but the lengths of chromosomes vary from one another, resulting in a series of tensors with different sizes for different chromosomes, especially on a genomic scale. Together with the high dimensionality of the contact matrix, especially at high resolutions, these methods are becoming inadequate for some issues. To address this problem, several strategies have been developed. Some directly utilize a series of input tensors with different sizes for chromosomes, such as the concatenation of the latent vectors for different input tensors in scDEC-Hi-C, while others split a contact matrix into a great number of sub-matrices with a fixed size by a divide-and-conquer strategy. Moving forward, with the emergence and development of the graph neural network, it was noticed that the contact matrix is symmetric and ultra-sparse at the single-cell level, which can be naturally regarded as the adjacency matrix of a graph, with nodes corresponding to bins and interaction frequencies being the weights of edges. Given this design, the imputation of scHi-C data can be regarded as how to recover the lost edges in a graph, which happens to be the well-known link prediction problem in graph neural networks, where the unseen edges can be predicted by leveraging other existing edges of the graph. Meanwhile, TAD structures can be considered the communities of nodes within a graph that have a stronger density of connections. Following this train of thought, some hypergraph or graph deep learning methods were developed, such as the hypergraph representation learning in Higashi for imputation on a genome scale and the graph VAE and hierarchical clustering with connectivity constraints in scCAFE for TAD identification. It is worth noting that the propagation of information from well-captured paths to under-captured paths during the training of the graph neural network contributes to the prediction of lost edges, which is the recovery of missing interactions. In addition, the aggregation of information from neighboring nodes benefits the capture of topological structures in the graph, facilitating the analysis of spatial organizations of chromatin, including TAD structures. In addition, the graph neural network is more suitable for application in multimodal analysis owing to the permission of multi-dimensional attributions of nodes and the existence of heterogeneous graphs. That makes the graph neural network highly attractive, especially with the continuous accumulation of simultaneous multimodal data beyond Hi-C data at the single-cell level, although its development is relatively insufficient.

## 6 Challenges and emerging trends

As an area of active research, the bioinformatic analysis of TADs on scHi-C data is expected to provide more knowledge beyond that on bulk Hi-C data. Undoubtedly, some challenges that have never been met at the bulk level will be encountered at the single-cell level. 1) The ultra-sparsity of scHi-C data. A Hi-C contact matrix for each cell is ultra-sparse due to the low sequencing depth, especially at higher resolutions. Coupled with noises, artifacts, and dropout events from experiments, the boundaries of TADs are utterly blurred, and their hierarchical patterns are even less visible. This poses great challenges to the identification of TAD structures and downstream analysis. 2) Heterogeneity of individual cells. At the single-cell level, individual cells within a population exhibit significant heterogeneity, even if they are of the same cell type. While examining the changes of TAD structures across cell populations, it is necessary to distinguish which differences are related to the normal evolutions of cellular functional states, such as the cell cycle and transcription status, and which are abnormal changes caused by different conditions of interest, such as the occurrence and development of diseases. The heterogeneity increases the difficulties for the bioinformatic analysis of TAD structures, especially differential analysis. 3) Lack of simultaneous multimodal data. Nowadays, researchers have started to introduce other omics data besides scHi-C data, such as bulk Hi-C data, scRNA-seq data, and scChIP-seq data, into the bioinformatic analysis of TADs. It is expected that the results can be improved by the fusion of information from multiple sources. The multimodal strategy is undoubtedly attractive, but in practice, there is a serious lack of multimodal data, especially simultaneous multimodal data, at the single-cell level due to experimental technologies, leaving bulk Hi-C data, which can only impose constraints on cell population as a whole, rather than on individual cells. Currently, it has been noticed that a few emerging bioinformatic tools are attempting to handle the non-simultaneous multimodal data, such as the generation of the scHi-C contact matrix from bulk Hi-C data using scRNA-seq as a guide signal in scGrapHiC (Murtaza et al., 2024) and the definition of cell subpopulations by integrating non-simultaneous scHi-C and scRNA-seq data into MUTI. This, in turn, further highlights the lack of simultaneous multimodal data for scHi-C data analysis. 4) Interplay between multi-scale architectures. The spatial organizations of chromatin on multiple scales, including A/B compartments, TADs, and loops, are not independent, but are nested and interactive. A/B compartments provide global active states, influencing the formation of TADs and loops. TADs confine the regulation to specific domains, giving a context for chromatin loops. The loops, in turn, are the specific means of implementing regulation within TADs. In addition, cell development and diseases are often associated with the disruptions on multiple scales. This makes it arduous to have an all-around view of organization changes and link them to biological processes by just relying on the analysis of TAD structures alone.

Considering the rapid development of bioinformatic analysis for TAD structures at the single-cell level and the challenges encountered at present, here is an arbitrary outlook for the emerging trends. 1) From the matrix-based neural network to GNN: benefiting from a cell number that can reach up to tens of thousands at the single-cell level, the matrix-based neural network has been widely used for analyzing scHi-C data, such as imputation. This kind of neural network can learn knowledge that is not easy to obtain for traditional methods, but it also faces a dilemma caused by the high dimensionality and ultra-sparsity of the scHi-C contact matrix, leading to the emergence of some networks that analyze small patches of the genome. Considering that the contact matrix is naturally suitable for being represented by graph-structured data due to its symmetry and ultra-sparsity, even on a genomic scale, the GNN seems promising for future applications, although it is not yet fully developed compared with the matrix-based neural network. 2) From scHi-C data to multimodal data: scHi-C data alone may have some limitations in the bioinformatic analysis of TAD structures, and the integration with other modal data may help refine this issue. For example, histone modification data can act as additional markers to demarcate TAD boundaries, reducing the adverse effects by ultra-sparsity and noises associated with scHi-C data. It is foreseeable that multimodal data analysis will play an important role in the near future, especially with the rapid development of experimental technologies and continuous accumulation of simultaneous multimodal data at the single-cell level. 3) From cell populations to temporal dynamics: the TAD structures of chromatin in the nucleus are temporally dynamic. Beyond the differential analysis of TADs between cell populations, tracking the dynamics may inform how TADs change over time. With the support of the scHi-C technology, the chromatin spatial interactions of a large number of individual cells at different time points are available. This allows us to develop bioinformatic tools to construct a trajectory for TAD structures during cell differentiation or disease progression, providing insights into their reorganizations and determining critical regulatory events. 4) From single-scale architecture to multi-scale architectures: the organizations of chromatin on multiple scales interact with each other to tune the spatial regulation of gene expressions. For example, a TAD within a compartment is more likely to contain actively transcribed genes, and a loop within the TAD can enhance the interaction between an enhancer and a promoter, which makes an investigation of TADs on a single scale far from enough. In the future, it is expected that a multi-scale analysis at the single-cell level may discover new spatial regulatory patterns by incorporating regulatory information from different scales.

## References

[B1] Arrastia M. V. Jachowicz J. W. Ollikainen N. Curtis M. S. Lai C. Quinodoz S. A. (2022). Single-cell measurement of higher-order 3D genome organization with scSPRITE. Nat. Biotechnol. 40(1), 64. 73. 10.1038/s41587-021-00998-1 34426703 PMC11588347

[B2] Berlivet S. Paquette D. Dumouchel A. Langlais D. Dostie J. Kmita M. (2013). Clustering of tissue-specific sub-TADs accompanies the regulation of HoxA genes in developing limbs. PLOS Genet. 9 (12), e1004018. 10.1371/journal.pgen.1004018 24385922 PMC3873244

[B3] Bintu B. Mateo L. J. Su J. H. Sinnott-Armstrong N. A. Parker M. Kinrot S. (2018). Super-resolution chromatin tracing reveals domains and cooperative interactions in single cells. Science 362 (6413), eaau1783. 10.1126/science.aau1783 30361340 PMC6535145

[B4] Bonev B. Cavalli G. (2016). Organization and function of the 3D genome. Nat. Rev. Genet. 17 (12), 772. 10.1038/nrg.2016.147 28704353

[B5] Chang L. Xie Y. Taylor B. Wang Z. N. Sun J. C. Armand E. J. (2024). Droplet Hi-C enables scalable, single-cell profiling of chromatin architecture in heterogeneous tissues. Nat. Biotechnol. 10.1038/s41587-024-02447-1 PMC12520981 39424717

[B6] Dang D. Zhang S.-W. Dong K. Duan R. Zhang S. (2024). Uncovering topologically associating domains from three-dimensional genome maps with TADGATE. Nucleic Acids Res. 53 (4). 10.1093/nar/gkae1267 PMC11879124 39727192

[B7] Dekker J. Rippe K. Dekker M. Kleckner N. (2002). Capturing chromosome conformation. Science 295(5558), 1306–1311. 10.1126/science.1067799 11847345

[B8] Dixon J. R. Selvaraj S. Yue F. Kim A. Li Y. Shen Y. (2012). Topological domains in mammalian genomes identified by analysis of chromatin interactions. Nature 485 (7398), 376–380. 10.1038/nature11082 22495300 PMC3356448

[B9] Flyamer I. M. Gassler J. Imakaev M. Brandão H. B. Ulianov S. V. Abdennur N. (2017). Single-nucleus Hi-C reveals unique chromatin reorganization at oocyte-to-zygote transition. Nature 544 (7648), 110–114. 10.1038/nature21711 28355183 PMC5639698

[B10] Han C. Xie Q. Lin S. (2020). Are dropout imputation methods for scRNA-seq effective for scHi-C data? Briefings Bioinforma. 22 (4). 10.1093/bib/bbaa289 PMC8293815 33201180

[B11] Hua D. Gu M. Zhang X. Du Y. Xie H. Qi L. (2024). DiffDomain enables identification of structurally reorganized topologically associating domains. Nat. Commun. 15 (1), 502. 10.1038/s41467-024-44782-6 38218905 PMC10787792

[B12] Li A. S. Zeng G. J. Wang H. Y. Li X. Zhang Z. H. (2023). DeDoc2 identifies and characterizes the hierarchy and dynamics of chromatin TAD-like domains in the single cells. Adv. Sci. 10 (20), e2300366. 10.1002/advs.202300366 PMC10369259 37162225

[B13] Li M. H. Yang Y. R. Wu R. C. Gong H. Y. Yuan Z. Wang J. X. (2025). SEE: a method for predicting the dynamics of chromatin conformation based on single-cell gene expression. Adv. Sci. 12 (8), e2406413. 10.1002/advs.202406413 PMC11848634 39778075

[B14] Li W. V. Li J. J. (2018). An accurate and robust imputation method scImpute for single-cell RNA-seq data. Nat. Commun. 9 (1), 997. 10.1038/s41467-018-03405-7 29520097 PMC5843666

[B15] Li X. Lee L. Abnousi A. Yu M. Liu W. Huang L. (2022). SnapHiC2: a computationally efficient loop caller for single cell Hi-C data. Comput. Struct. Biotechnol. J. 20, 2778–2783. 10.1016/j.csbj.2022.05.046 35685374 PMC9168059

[B16] Li X. Zeng G. J. Li A. S. Zhang Z. H. (2021). DeTOKI identifies and characterizes the dynamics of chromatin TAD-like domains in a single cell. Genome Biol. 22 (1), 217. 10.1186/s13059-021-02435-7 34311744 PMC8314462

[B17] Lieberman-Aiden E. van Berkum N. L. Williams L. Imakaev M. Ragoczy T. Telling A. (2009). Comprehensive mapping of long-range interactions reveals folding principles of the human genome. Science 326 (5950), 289–293. 10.1126/science.1181369 19815776 PMC2858594

[B18] Liu E. Lyu H. Liu Y. Fu L. Cheng X. Yin X. (2024). Identifying TAD-like domains on single-cell Hi-C data by graph embedding and changepoint detection. Bioinformatics 40 (3), btae138. 10.1093/bioinformatics/btae138 38449288 PMC10960928

[B19] Liu Q. Zeng W. Zhang W. Wang S. Chen H. Jiang R. (2022). Deep generative modeling and clustering of single cell Hi-C data. Briefings Bioinforma. 24 (1), bbac494. 10.1093/bib/bbac494 36458445

[B20] Murtaza G. Butaney B. Wagner J. Singh R. (2024). scGrapHiC: deep learning-based graph deconvolution for Hi-C using single cell gene expression. Bioinformatics 40 (Suppl. ment_1), i490–i500. 10.1093/bioinformatics/btae223 38940151 PMC11256916

[B21] Nagano T. Lubling Y. Stevens T. J. Schoenfelder S. Yaffe E. Dean W. (2013). Single-cell Hi-C reveals cell-to-cell variability in chromosome structure. Nature 502 (7469), 59–64. 10.1038/nature12593 24067610 PMC3869051

[B22] Nagano T. Lubling Y. Varnai C. Dudley C. Leung W. Baran Y. (2017). Cell-cycle dynamics of chromosomal organization at single-cell resolution. Nature 547 (7661), 61–67. 10.1038/nature23001 28682332 PMC5567812

[B23] Peng T. Zhu Q. Yin P. H. Tan K. (2019). SCRABBLE: single-cell RNA-seq imputation constrained by bulk RNA-seq data. Genome Biol. 20, 88. 10.1186/s13059-019-1681-8 31060596 PMC6501316

[B24] Phillips-Cremins J. E. Sauria M. E. G. Sanyal A. Gerasimova T. I. Lajoie B. R. Bell J. S. K. (2013). Architectural protein subclasses shape 3D organization of genomes during lineage commitment. Cell 153 (6), 1281–1295. 10.1016/j.cell.2013.04.053 23706625 PMC3712340

[B25] Rao S. S. Huntley M. H. Durand N. C. Stamenova E. K. Bochkov I. D. Robinson J. T. (2014). A 3D map of the human genome at kilobase resolution reveals principles of chromatin looping. Cell 159 (7), 1665–1680. 10.1016/j.cell.2014.11.021 25497547 PMC5635824

[B26] van Dijk D. Sharma R. Nainys J. Yim K. Kathail P. Carr A. J. (2018). Recovering gene interactions from single-cell data using data diffusion. Cell 174 (3), 716–729.e27. 10.1016/j.cell.2018.05.061 29961576 PMC6771278

[B27] Wang F. Lin J. Alinejad-Rokny H. Ma W. Meng L. Huang L. (2024). Unveiling multi-scale architectural features in single-cell Hi-C data using scCAFE. bioRxiv 2024, 2009.2010. 10.1101/2024.09.10.611762 PMC12199332 40270467

[B28] Xie Q. Han C. Jin V. Lin S. (2022). HiCImpute: a Bayesian hierarchical model for identifying structural zeros and enhancing single cell Hi-C data. PLOS Comput. Biol. 18 (6), e1010129. 10.1371/journal.pcbi.1010129 35696429 PMC9232133

[B29] Ye Y. S. Zhang S. H. Gao L. Zhu Y. Q. Zhang J. (2023). Deciphering hierarchical chromatin domains and preference of genomic position forming boundaries in single mouse embryonic stem cells. Adv. Sci. 10 (8), e2205162. 10.1002/advs.202205162 PMC10015865 36658736

[B30] Zeng Q. Xiang G. Zhang Y. Hardison R. C. Li Q. (2024). JOnTADS: a unified caller for TADs and stripes in Hi-C data. bioRxiv. 10.1101/2024.11.06.622323

[B31] Zhang R. Zhou T. Ma J. (2022). Multiscale and integrative single-cell Hi-C analysis with Higashi. Nat. Biotechnol. 40 (2), 254–261. 10.1038/s41587-021-01034-y 34635838 PMC8843812

[B32] Zheng J. H. Yang Y. D. Dai Z. M. (2024). Subgraph extraction and graph representation learning for single cell Hi-C imputation and clustering. Briefings Bioinforma. 25 (1). 10.1093/bib/bbad379 PMC10691963 38040494

[B33] Zheng Y. Shen S. Keleş S. (2022). Normalization and de-noising of single-cell Hi-C data with BandNorm and scVI-3D. Genome Biol. 23 (1), 222. 10.1186/s13059-022-02774-z 36253828 PMC9575231

[B34] Zhou J. Ma J. Chen Y. Cheng C. Bao B. Peng J. (2019). Robust single-cell Hi-C clustering by convolution- and random-walk–based imputation. Proc. Natl. Acad. Sci. 116(28), 14011–14018. 10.1073/pnas.1901423116 31235599 PMC6628819

[B35] Zhou Y. Li T. Choppavarapu L. Fang K. Lin S. Jin V. X. (2024). Integration of scHi-C and scRNA-seq data defines distinct 3D-regulated and biological-context dependent cell subpopulations. Nat. Commun. 15 (1), 8310. 10.1038/s41467-024-52440-0 39333113 PMC11436782

